# Twinning Partnership Network: A Learning and Experience-Sharing Network Among Health Professionals in Rwanda to Improve Health Services

**DOI:** 10.9745/GHSP-D-23-00280

**Published:** 2024-10-29

**Authors:** Celestin Gasana, R. Taylor Williamson, Ursin Bayisenge, Jean Claude Rukundo, Modeste Gashayija, Edward Kamuhangire, Corneille Ntihabose, Joy Atwine, Theophile Nsengiyumva, Solange Hakiba, Bienvenu Niyongabo

**Affiliations:** a CARE Canada; formerly of RTI International, Winnipeg, Canada.; b Abt Global; formerly of RTI International, Rockville, MD, USA.; c Independent Consultant/RTI International, Kigali, Rwanda.; d Ministry of Health, Kigali, Rwanda.; e Management Sciences for Health; formerly of Palladium, Kigali, Rwanda.; f Independent consultant; formerly of Palladium, Kigali, Rwanda.; g Palladium, Kigali, Rwanda.

## Abstract

We describe the development, implementation, and evaluation of a novel twinning approach: the Twinning Partnership Network (TPN). Twinning is a well-known approach to peer learning that has been used in a variety of settings to build organizational capacity. Although twinning takes many forms, the heart of the approach is that institutions with shared characteristics collaborate via sharing information and experiences to achieve a specific goal. We adapted a twinning partnership strategy developed by the World Health Organization to create a network of like-minded health institutions. The key innovation of the TPN is the network, which ensures that an institution always has a high-performing peer with whom to partner on a specific topic area of interest. We identified 10 hospitals and 30 districts in Rwanda to participate in the TPN. These districts and hospitals participated in a kickoff workshop in which they identified capacity gaps, clarified goals, and selected twinning partners. After the workshop, districts and hospitals participated in exchange visits, coaching visits, and virtual and in-person learning events. We found that districts and hospitals that selected specific areas and worked on them throughout the duration of the TPN with their peers improved their performance significantly when compared with those that selected and worked on other areas. Accreditation scores improved by 5.6% more in hospitals selecting accreditation than those that did not. Districts that selected improving community-based health insurance coverage improved by 4.8% more than districts that did not select this topic area. We hypothesize that these results are due to senior management’s interest and motivation to improve in these specific areas, the motivation gained by learning from high-performing peers with similar resources, and context-specific knowledge sharing from peer hospitals and districts.

## Abstract (precis)

Twinning and peer-to-peer learning networks can play a pivotal role in building strong institutions and improving performance, leveraging both local and external expertise where learning and collaboration occur both within and beyond local contexts.

## Teaser

**Key Findings:**

- A twinning network can produce significant improvements in organizational, managerial, and clinical performance across a diverse range of indicators.
- A twinning network can provide positive empowerment and motivational effects but requires the right combination of senior leadership involvement, preexisting capacity within the twinning network, and opportunities for sharing knowledge.
- The twinning partnership network approach, particularly in the context of Rwanda, exemplifies how such networks can be effectively used to strengthen health systems and drive sustainable development.

**Key Implications:**

- In settings with organizational and clinical capacity that varies across similar types of institutions, policymakers should consider using twinning networks to unlock capacity and skills that already exist and place peer institutions in teaching roles rather than using external technical “experts.”
- For twinning networks to be successful, donors and program implementers should focus on several key factors, such as institutions having the ability to set their own learning goals that are relevant and tailored to their specific needs, having high-performing peer institutions that can serve as effective mentors and models, and having participating institutions that can dedicate adequate time and resources to improving key performance indicators.

## BACKGROUND

Rwanda has made strong health gains over the last 2 decades, having achieved most of the Millennium Development Goals as a result of increased population coverage and higher-quality health services that emphasize quality of life, health system performance, and social determinants of health.^1^^–^^3^ To support these health improvements, Rwanda decentralized governance functions in phases.^4^

The first phase of decentralization (2001–2005) aimed at creating “democratically elected and community development structures.” The second phase design (2006–2010) followed the nation’s 2005 territorial restructuring, which decreased the number of districts from 106 to 30 and combined health and administrative districts. The goal of this Rwanda Decentralization Plan was to achieve equal political, economic, and social development across the country by enhancing public engagement in decision-making and governance.^4^

To align with Rwanda’s National Decentralization Plan, the Ministry of Health (MOH) outlined major principles to follow for decentralizing the Rwandan health system and improving the geographic and financial accessibility of equitable and affordable quality health services for all Rwandans.^5^^–^^9^ In service to these principles, the MOH established the district as the basic operational unit of the health system.^4^ As of 2023, the Rwanda health system operates in accordance with its decentralization framework and policy (Box 1, Figure).^8^

**Figure:**
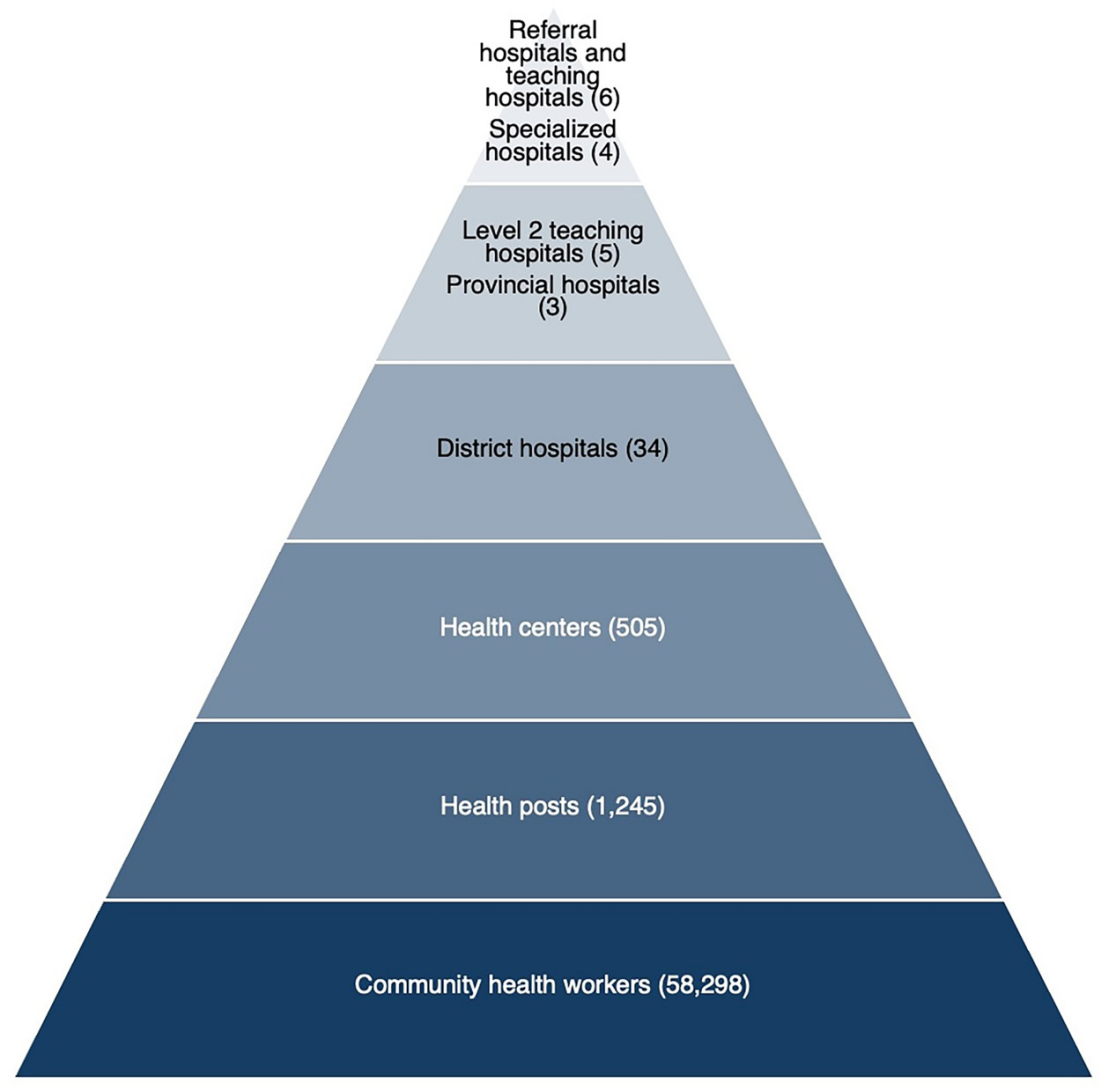
Rwanda Health System Source: Rwanda Ministry of Health.

BOX 1 Roles and Responsibilities in the Rwandan Health System

**District health management team (DHMT):** As described in the district health system reorganization guideline from a managerial perspective, the DHMT’s main role is to oversee health services’ planning, management, monitoring, coordination, financial and resource oversight, and regulation, as well as enhance local community involvement in the delivery and management of health services.

**District health unit (DHU):** As part of the administrative district organizational structure, the DHU coordinates the provision of health services while maintaining oversight of the district health system and is responsible for organizing, carrying out, and keeping track of the district’s health activities. The DHU also provides technical oversight of decentralized institutions and organizations, whether public, private, or nonprofit.

**Hospitals:** As defined in the health services package, hospitals’ primary responsibilities include supporting preventive and promotional initiatives within the catchment area and providing curative and rehabilitative treatment, including for referred cases. In Rwanda, hospitals are classified into 3 groups based on the services they offer (Figure):

1. District hospitals offer simple surgeries, preventative care, and treat referred cases and are the entry point to the hospital system.
2. Provincial and Level 2 teaching hospitals have more specialized inpatient and emergency care than district hospitals and offer health professional training.
3. Referral hospitals, teaching hospitals, and specialized hospitals carry out research and offer more sophisticated, specialized care than either district or provincial hospitals. They serve as referral hospitals for the provincial and district hospitals and educate and train medical personnel.

Through decentralization, Rwanda’s health system has seen notable improvements, particularly in increasing health insurance coverage. Community-based health insurance (CBHI) now covers more than 83% of the population, a significant increase from the 27% coverage reported in 2004.^10^ This expansion has contributed to enhanced health outcomes, such as a 77% reduction in mortality rates for children younger than 5 years from 196 deaths per 1,000 live births to 45 and a substantial decrease in maternal mortality from 1,071 deaths per 100,000 live births in 2000 to 203 in 2020.^10^ Additionally, health services have become more geographically accessible and coordination and involvement of health sector actors in managing and controlling health interventions has improved, leading to improved health outcomes across the country.^6^^,^^10^^–^^13^

However, despite these achievements, disparities in capacity and performance persist across districts and hospitals. To address these variations and further strengthen the health system, a twinning approach was implemented. Twinning facilitates peer-to-peer learning and capacity-building by pairing higher-performing districts and hospitals with those needing improvement. This approach leverages existing expertise within the country, promoting sustainable development and continuous improvement in health care delivery.

Twinning facilitates peer-to-peer learning and capacity-building by pairing higher-performing districts and hospitals with those needing improvement.

The American International Health Alliance defines twinning as “partnerships that link 2 entities with shared characteristics to achieve a common goal.”^14^ The World Health Organization defines partnership as “a collaborative relationship between 2 or more parties based on trust, equality and mutual understanding for the achievement of a specified goal.”^15^ Argaw et al. said that a twinning partnership “follows a ‘win-win’ mutual relationship and jointly understood principles,” in which institutions partner in a spirit of mutual learning; problem-solving; and sharing information, experiences, and sometimes resources.^16^

Twinning partnerships have had success in various forms, including those of towns on different continents, businesses in related industries, research institutions, HIV programs around the world, and health facilities dealing with similar challenges.^17^^–^^19^ These institutions have successfully collaborated to learn from one another and solve problems, as well as share knowledge, expertise, and resources.

Successful twinning partnerships typically include the following crucial components:

- Professional exchanges and mentoring for efficient sharing of information, knowledge, and technology
- Voluntary contributions of knowledge, time, skills, and resources (e.g., equipment and reference materials)
- Peer relationships that benefit both parties^16^

Where twinning partnerships have been implemented to address a specific issue or challenge, they have benefited the twinned institutions in the ways identified in Table 1. The literature also showed some challenges for twinning partnerships. Argaw et al. identified the challenges included having unclear expectations and understanding of the goals of twinning, the impression that twinning is a waste of resources, especially when costly travel and per diem are involved, and poor transparency of staff selection for participation in twinning events.^16^ However, Cadée noted that balancing power dynamics in twinning partnerships is difficult, especially in cases of disparate performance. Developing true reciprocity, where both partners benefit, is “a process that needs to be given time.”^20^

**TABLE 1.** Twinning Partnership Examples

**Goal and Description**	**Outcome**
Twinning partnerships among 8 districts in Ethiopia in 2018 to improve performance of district health systems in Ethiopia.^16^	High-performing primary health care units saw their mean score rise from 17.96 to 23.32. Woreda management standards improved from 6.42 to 8.15, and model villages increased from 11.58 to 20.85. Overall, district health system performance improved from 50.97 to 72.07.
The Ministry of Health in Tonga and St. John of God Hospital in Ballarat, Australia, had a 25-year twinning partnership that encouraged open discussion and collaboration.^17^	Enhanced skills transfer, learning, and improved health care practices.
Twenty-six organizations that focused on HIV support in Canada partnered with HIV projects in several low- and middle-income countries to strengthen capacity of both HIV-focused organizations and projects.^18^	Identification of communication and cultural barriers, improved programming, new skills in developing partnership agreements, integration of international perspectives into routine work, and improved information exchange.
ANEMO partnered with the African Palliative Care Association from Uganda to strengthen ANEMO's human and institutional capacity to implement, monitor, and evaluate programs.^19^	ANEMO developed a new governance structure, enhanced strategic planning, and improved professional practices. ANEMO also incorporated effective palliative care methods learned from Uganda, which enhanced their ability to manage and sustain HIV programs.

Abbreviation: ANEMO, Associação Nacional dos Enfermeiros de Moçambique.

### Twinning Partnerships in Rwanda

In Rwanda, twinning partnerships have existed under different names and forms. For example, the longest-lasting twinning partnership is Jumelage, between Rwanda and Rhineland-Palatinate, a state of Germany. In this program, the 2 governments collaborate to facilitate professional interaction between municipalities and institutions across both countries.^21^

The Rwanda Human Resources for Health (HRH) program used twinning as its core principle in training and developing health professionals by establishing partnerships with U.S. institutions for teaching. Rather than sending Rwandan professionals to the United States for specialty and subspecialty training, HRH hired faculty from 23 U.S. institutions, such as Brigham and Women’s Hospital, the University of Maryland School of Nursing, and the Harvard School of Dental Medicine, to work in Rwanda. U.S. faculty collaborated with Rwandan academics at universities and clinical teaching sites.^22^ This twinning partnership aimed to transmit skills to Rwandan faculty members, who would then mentor new Rwandan academics and train future health professionals. In a survey assessing the HRH twinning program, 89% of Rwandan faculty and 71% of U.S. faculty reported setting joint goals with their twin, indicating strong alignment in goal setting. Satisfaction with the twinning experience was high, with 93% of Rwandan faculty and 59% of U.S faculty expressing satisfaction.^23^

In the education sector, the Rwanda Education Board, in collaboration with the Flemish Association for Development Cooperation and Technical Assistance, initiated professional learning networks, an adapted form of twinning that includes more than 2 relationships. These networks are part of a larger peer-to-peer learning network between head teachers that encourages learning and collaboration, in which school leaders share their knowledge and experiences. The professional learning networks are an opportunity to offer a better perspective on school leadership and management and foster learning, engagement, and collaboration among school leaders.^24^

These forms of twinning have different purposes, and the results vary. For example, the twinned governments assessed Jumelage on the number of projects developed and implemented together, as well as the amount of funds invested in the partnership. The Government of Rwanda established the HRH twinning program as a more affordable alternative to sending students abroad to study medical specialties. Through this partnership, the University of Rwanda benefits from experts from U.S. institutions, and the twinning program’s effectiveness is assessed through the number of specialists received from these institutions.^23^ The professional learning networks support head teachers to build and maintain relationships, increase performance, provide a sense of strategic vision, and improve working environments and motivation among network members.^24^^,^^25^

## TWINNING PARTNERSHIP NETWORK IN RWANDA

### Structure

We designed the Twinning Partnership Network (TPN) using the World Health Organization’s Twinning Partnerships for Improvement (TPI) model as a framework and adapted some aspects of its 6-step improvement cycle, including partnership development, needs assessment, gap analysis, and action planning.^15^ The TPN was developed with funding from the Rwanda Integrated Health System Activity (RIHSA), a 3-year, U.S. Agency for International Development (USAID)-funded project.

The TPN structure differs from the TPI model in several key aspects. The TPI model, as well as most twinning activities, typically involves a dyad, where an institution partners with another. Each institution in this dyad learns from the other in areas of mutual interest. In contrast, the TPN employs a network approach. Instead of a simple dyadic relationship, the TPN establishes a network where Hospital X learns from Hospital Y, and Hospital Y, in turn, learns from Hospital Z, creating a more interconnected learning network.^15^ District health management teams (DHMTs) or hospitals that wish to learn in 1 area are categorized as “rising,” while those that are performing well and can act as mentors are categorized as “shining.” A “rising” DHMT or hospital is paired with a “shining” DHMT or hospital to facilitate learning. “Rising” and “shining” DHMTs and hospitals are determined based on existing metrics, such as accreditation scores, health outcomes, and service indicators. In this model, a DHMT or hospital can be “shining” in a topic area and “rising” in another, resulting in a web of twinning relationships.

The TPN employs an interconnected learning network approach.

Second, the TPI does not specify activities; rather, it leaves institutions to develop their own activities. The TPN, in contrast, recommends specific actions to build relationships between DHMTs and hospitals. These actions, such as coaching visits, exchange visits, and learning events, described in the next section, help “shining” DHMTs and hospitals teach what they do well, “rising” DHMTs and hospitals learn, and study team facilitators to track progress (Box 2).

BOX 2 6 Cs of the Rwanda Twinning Partnership Model

- **Collaboration** between institutions and key actors in health systems
- **Commitment** to learning, performance improvement, and supporting one another
- **Connection** that promotes mutual support and learning
- **Cooperation** between twinned institutions to facilitate change
- **Coaching** to connect twinned institutions and keep teams focused on results
- **Continuous learning** that is locally sustainable and promotes resilience in the health system

Finally, the TPI does identify some potential costs, such as visits, but does not identify what funding is needed. The TPN was funded by USAID RIHSA, which covered study team labor, per diems, and travel costs for TPN participants and the study teams for exchange visits and workshops, and all workshop costs. Districts and hospitals contributed staff time and space for exchange visits and some learning visits. The MOH also provided oversight and management expertise to the TPN.

### Design Process

#### Co-design

To start the TPN, RIHSA and the MOH conducted co-design workshops with 10 hospitals that were selected; all 30 DHMTs were included in the TPN. The participating hospitals were identified based on the needs and MOH guidance. This selection process involved reviewing health system data and consulting with the MOH (Supplement 1 lists selection criteria). The workshops aimed to refine the twinning approach to meet local needs and structures, establish TPN goals, create network-level performance indicators to measure success, and identify data sources for those indicators (Table 2). This step enabled stakeholders to customize the twinning approach to align with MOH priorities.

**TABLE 2.** Key Events in the Twinning Partnership Network Development Process, Rwanda, August 2021–February 2023

**Event**	**Purpose**	**Participants**	**No. of events**
Co-design	Refine and customize the approach to Rwandan context	MOH, hospitals, DHMTs	2 (1 for DHMTs and 1 for hospitals)
Twinning launch	Launch the partnerships and develop twinning partnership plans for each participating institution	DHMTs, hospitals, MOH, USAID-RIHSA	1 workshop (split into 4 sessions based on the number of participants, 10 DHMTs/hospital per session)
Coaching (both virtual and in person)	Provide coaching and review progress, challenges as well as document best practices and lessons learned	DHMTs and hospital	3 coaching sessions per each member (1 virtual and 2 in-person coaching)
Learning and experience-sharing	Share experience across the wide TPN	MOH, DHMTs and hospitals	4 virtual workshops organized (topics of interest identified by TPN members)
Provincial learning events workshop	DHMTs and hospitals in the same province to meet, learn, and share experience	DHMTs, hospitals, MOH	4 workshops (1 per province)

Abbreviations: DHMT, district health management teams; MOH, Ministry of Health; RIHSA, Rwanda Integrated Health System Activity; TPN, twinning partnership network; USAID, U.S. Agency for International Development.

#### Twinning Launch Workshops

The MOH and RIHSA held launch workshops to introduce DHMTs and hospitals to twinning, identify critical challenges, and prioritize performance improvement issues. During these workshops, facilitators guided each DHMT and hospital team through a structured process to identify and rank 2–5 priority areas or challenges they wanted to tackle through the twinning partnership (Supplement 2 Table S1 and Table S2). They recorded each priority on separate sticky notes. Additionally, each team identified 1–3 areas where they were performing well and could offer expertise to others. These priorities and strengths were displayed on a whiteboard, backed by data to show their current standings.

Facilitators then assisted in matching “rising” DHMTs or hospitals with “shining” DHMTs or hospitals based on the identified priorities and strengths. This matching process ensured that specific skills available in a team could meet the needs of another team seeking to improve in that area. Each team established objectives for their twinning partnership, including baselines and milestones. The workshops culminated in the development of detailed twinning partnership plans, where DHMTs and hospitals committed to supporting one another both remotely and in person. This structured approach provided a clear framework for addressing performance challenges and leveraging each partner’s strengths for mutual benefit.

#### Coaching

Following the launch workshops, the study team conducted a coaching session for each of the DHMTs and hospitals in the TPN, focusing on refining the twinning objectives and finalizing milestones and targets to achieve during the partnership. About 2–3 months after the first coaching visit, the study team conducted a second coaching session for each TPN member to review progress, challenges, and solution strategies. The study team conducted a third and final coaching session about a year later for each member to review progress and achievements, as well as document best practices and lessons learned.

#### Experience-Sharing

Through the TPN, DHMTs and hospitals had the opportunity to lead virtual learning events on hospital accreditation, CBHI, and antenatal care. During these events, DHMTs and hospitals shared their experiences on performance improvement processes and new approaches and initiatives. Provincial learning events, facilitated by the study team and the MOH, allowed high-performing DHMTs and hospitals to share their experiences and progress journeys, outline success factors and best practices for others, and describe new ideas and strategies to achieve higher performance.

#### Exchange Visits

Before each exchange visit, a study team member worked with the “shining” DHMT or hospital to develop the visit agenda, including presentations and tours. During exchange visits, 3 staff from “rising” DHMTs or hospitals traveled to visit their “shining” twins that had demonstrated good performance. Staff from the “shining” DHMTs or hospitals gave presentations on the area of focus, gave tours of their facilities, facilitated discussions, and shared information, observations, and experiences with colleagues in similar roles to discuss challenges and potential solutions. A study team member attended the exchange visit to supervise, but all presentations, facilitation, and tours were conducted by the staff of the “shining” DHMT or hospital.

During exchange visits, staff from “rising” DHMTs or hospitals traveled to visit their “shining” twins to hear presentations on the area of focus, go on tours of the facilities, participate in facilitated discussions, and learn information.

At the completion of each visit, the visiting “rising” team developed an improvement plan that included actions and steps to implement once they returned to their respective workplaces. The benefits for the “shining” institutions included recognition for their best practices, the opportunity to refine their own processes through sharing and feedback, the establishment of stronger networks within the health system, and learning from others in the network. Because the TPN is not structured as a dyad, the “shining” hospitals and DHMTs in an area could be “rising” in another, allowing for reciprocal learning and continuous improvement across different domains.

## METHODS

Results presented include both quantitative data and secondary data from government reports (e.g., MOH results on accreditation and the Rwanda Social Security Board’s CBHI data). Qualitative data were collected from in-depth interviews conducted during the final in-person coaching visits. The interviewers (2 project consultants) used a discussion guide to conduct the in-depth interviews that focused on 4 key areas: improvements the DHMT or hospital made because of their participation in the TPN, challenges that prevented them from achieving their twinning partnership plan, lessons for improving the TPN, and their ideas for next steps for the TPN. The discussion guide can be found in Supplement 3.

The project consultant team conducted group interviews with 3 representatives from each of the 30 DHMTs and 10 hospitals in the TPN, for a total of 120 interviewees. The study team made all efforts to interview participants from the DHMTs and hospitals who had been involved in the TPN since the launch workshops. However, given staff turnover and availability, some interviewees did not participate in all the twinning activities. At least 1 interviewee from each of the DHMTs and hospitals had participated in the TPN since the launch workshop. The project consultants conducted interviews in Kinyarwanda, but interviewers took detailed written notes in English. Participants gave their informed consent.

In-depth interviews were not recorded; instead, to ensure thoroughness and capture the participants’ perspectives as accurately as possible, interviewers took detailed notes during the discussions, which were then transcribed into Microsoft Word. Although the discussion guide consisted of only 4 themes, the 40 interviews yielded 53 pages of notes.

As a result, the study team coded the qualitative analysis by hand, identifying critical themes, counting the number of times those themes were mentioned, and highlighting representative quotes for those themes. This method ensured that the analysis was thorough and reflective of the participants’ perspectives.

Secondary data consisted of 2 main types. First, the MOH shared accreditation results for all hospitals in Rwanda in 2021, 2022, and 2023 with the project team. To evaluate hospital performance, the project team used accreditation scores because they are a widely used metric in Rwanda, are closely tracked by the MOH because the accreditation studies are conducted every year, and many DHMTs (for their district hospitals) and hospitals selected it as a priority area. Accreditation surveys were conducted every April and May in all hospitals in Rwanda using a standardized tool that was comparable across hospitals and years.^26^ Rwandan hospitals have 3 levels of accreditation. For this analysis, we focused on level 2 accreditation, as that level was the goal for most of the hospitals in the TPN. Using this data set, we compared performance between hospitals that selected accreditation as a priority area and those that did not select accreditation to understand whether focusing on accreditation through the TPN improved performance compared to other areas.

Second, districts provided the project team with access to CBHI enrollment data for 2021, 2022, and 2023. We used these data to compare DHMTs that chose CBHI enrollment as their priority area against those that chose another priority area. CBHI enrollment data were collected from financial institutions and agents (Umurenge SACCO, Irembo, Mobile money agent, Mobicash) that received household contributions through an online system managed by the Rwanda Social Security Board.^27^ Every Friday, the Rwanda Social Security Board provided a weekly progress update disaggregated by district so that each district could track the enrollment rate based on contributions received.

In addition, we calculated the percentage of institutions that showed improvement, remained the same, and declined. We used the right-tailed z-test to compare mean performance scores before and after the intervention. The results of these analyses, including *P*-values, are presented to highlight statistically significant changes.

### Ethical Approval

The MOH approved this, and hospital administration and leadership were informed of this activity.

## RESULTS

### Twinning as a Driver in Improving Hospital Accreditation

Our results show that hospitals improved accreditation scores, strengthened service quality standards, and reduced stock-outs of medical supplies. The Rwanda Hospital Accreditation Standards outline 3 levels of accreditation achievements: (1) the policies, procedures, protocols, and plans have been developed and communicated that describe the expected quality of care/services to be provided; (2) the processes (described in the policies, procedures, protocols, and plans) are implemented in a consistent way; and (3) there are data to confirm successful risk-reduction strategies and continued improvement.

The results from an independent accreditation evaluation for 2021 and 2023 showed that the 19 hospitals that were TPN members and selected improving accreditation scores as their priority area increased their level 2 accreditation performance score^28^ by 7.8% (Supplement 4 Table S1), whereas the 19 hospitals that were not TPN members increased their level 2 accreditation performance score by only 2.2% (Supplement 4 Table S2) (*P*<.001) (unpublished data). The 19 hospitals that selected accreditation as their focus area had lower initial scores than the 19 hospitals that were not TPN members. Their lower initial scores likely led to their selection of accreditation as their focus area, as they had some extrinsic motivation to improve their accreditation scores.

Among hospitals that selected accreditation as their priority area, 73.7% increased performance, but 26.3% declined in performance. Of the hospitals that did not select accreditation as their priority, 63.1% increased performance, and 36.9% decreased performance. There is no hospital that maintained the same performance score over that period.

In addition, for 6 hospitals that selected a priority area other than improving their accreditation scores, their average improvement in accreditation scores was greater than for those that chose to address accreditation scores (Supplement 4 Table S3). This finding is driven by exceptionally low scores in 2021 for Kibagabaga and Kabutare hospitals, which had the 2 lowest accreditation scores in 2021, and their subsequent improvement to scores closer to the average in 2023.

### Twinning as a Driver in Improving Health Insurance Coverage

For CBHI, we compared coverage data in all 30 districts for the same period in the year (March 23, 2021, to March 23, 2023). The 8 districts that chose CBHI enrollment as their focus area showed an increase in coverage of 6.6% (Supplement 4 Table S4), compared to a 3.0% increase in districts that did not choose it as their priority area (Supplement 4 Table S5)^26^ (*P*<.005). For districts that chose CBHI enrollment as their focus area, these figures represent a 35% decline in the uninsured population, compared to a 22% decline among districts that did not choose CBHI enrollment as their focus area.

### Twinning as a Tool to Build Strong Collaboration and Networks

The TPN is an opportunity for health professionals to formally establish collaboration and communication with other health professionals. Before the TPN, collaboration between staff from different districts and hospitals was ad hoc and informal. In interviews, district health staff noted how the TPN strengthened relationships to help them improve performance.

*Through the twinning partnership, we have expanded our networks with colleagues from other districts, we communicate officially, and we are not shy to ask advice and guidance from our fellow health professional[s] whom we know are performing better in an area where we are still struggling.* —District director of health

*Learning from others was beneficial for us, it helped us to get rid of our routine way of doing things. We had a chance to learn new skills and strategies from our twin partner, and we are applying them, and we are witnessing improvement.* —District director of health

The TPN also created synergy between departments within a district or hospital, encouraging open discussion between health professionals and managers.

*There [are] no other learning opportunities that bring together staff and their leaders to observe a good practice that can help to solve a known problem.* —Hospital quality improvement officer

*As a private clinic, the exchange visit to [our twin partnered hospital] was an eye-opener to our leaders and staff who work in a small private health facility. The way their laboratory was organized has opened our senior management’s eyes; they witnessed everything that we have been advocating in order to improve laboratory services in our clinic, and from there, the leadership is committed to implement necessary changes that will result in improved service.* —Hospital laboratory manager

### Twinning as an Opportunity for Learning and Experience Sharing

TPN members also said it provided them with an opportunity to learn from others, know what approaches and strategies have worked in similar settings, and identify best practices to apply in their institutions.

TPN members said the network provided them with an opportunity to learn from others, know what approaches and strategies have worked in similar settings, and identify best practices to apply in their institutions.

*Learning from others always helps to connect with the reality, it is also a kind of self-accountability when a team is learning from others who have succeeded or made good progress in a certain area; they realize their weaknesses, things they are doing in [the] wrong way, and eventually learn how others have overcome the similar challenges they are facing.* —District director of health

*For me, twinning is the only channel we can use, as health care workers, to understand really all facets of the accreditation, because hearing and observing new approaches and strategies from other hospitals unlock[s] misunderstandings and give[s] us wide perspectives and a better understanding of what exactly is required for some of the accreditation standards.* —Director of nursing

*The Twinning Partnership[s] Network is a safe and effective channel to challenge and change the status quo. Even well-performing districts still have a lot to learn from other districts. Twinning partnerships open up people’s mind[s] to [an] extent they can only know by visiting and seeing how others do their things.* —Health promotion officer

*Before pairing and visiting [another district], we were not sure that we [would] learn anything new from them. However, during our exchange visit, we were very surprised with their strategies. Learning from this particular district was a great opportunity for us, because [its] population’s characteristics and living conditions are similar [to ours]; both being rural districts, and [the] source of income for the households are very similar. We are progressing very well to achieve our twinning objective, and strategies being used are those learnt during the exchange visit.* —District director of health

### Twinning as an Approach to Institutionalize Structures and Systems

Through the TPN, DHMTs and hospitals learned how to operationalize existing structures to deliver their mandated goals, as well as establish new ones when they were missing. For private facilities, the twinning approach was an opportunity to understand accreditation requirements, both in staff roles and responsibilities, and establish new structures, such as committees, required by the Rwanda hospital accreditation standards.

*For us, as a private hospital, things are different from what is happening in public hospitals—[the] accreditation process is new in the private hospitals in Rwanda. Therefore, after the exchange visit conducted at [our twinning partner hospital], [our] hospital administration recognized the role of quality improvement officer, which was not the case before we joined the [TPN]. Now, my time is 100% allocated to supporting the quality improvement in the hospital, supporting the hospital in developing contract[s], [and] leading the development of policies and standard procedures, as well as other tasks related to quality improvement.* —Quality improvement officer

*Right after the visit to [our twinning partner district], one of the new initiative[s] was to make the existing accreditation committee more dynamic and functional. In collaboration with the hospital leadership, all committees were operationalized by appointing staff in leadership positions to head these committees, and [hospital leadership] emphasized … accountability and linked it to performance appraisal.* —District director of health

The TPN also motivated health professionals to achieve performance goals, especially when there was buy-in and interest from senior leadership.

*For us, the governor of our province is constantly … asking us where we are with the implementation of what we learned through … from the exchange visit we conducted in one of the best district performer[s] in [the] community-based health insurance area, and this push[es] us to keep working hard and deliver, because it has now become like a formal commitment that we have to deliver.* —District director of health

*The actual involvement and commitment of the hospital [director general] is very promising, we are quite sure that achieving Level 2 of hospital accreditation is now possible.* —District director of health

*Involvement of the vice mayor allowed the district executive committee to better understand the role of the district in supporting our hospital in their accreditation journey … especially for areas which require additional budget or advocacy. As [a] result, now the district executive committee is advocating for the [installation of a wall around the hospital] as one of the area[s] that affect[s] the performance of our hospital in the accreditation process.* —District director of health

### Twinning as an Opportunity to Self-Analyze

Being part of the TPN provided members with the opportunity to conduct a deep self-analysis, identifying areas in which they were underperforming and analyzing and documenting the causes and factors influencing that underperformance. This self-analysis gave them opportunities to explore where skills and best practices exist locally and establish a partnership that aims at learning and sharing knowledge, skills, and experiences.

*[The] twinning partnership allowed us to take time to analyze the gaps and areas that we need to improve. For instance, when we look at our performance in the first antenatal care visits coverage and looking at data from our neighboring district … we asked ourselves, what went wrong with us? How come … we are underperforming? What can we do differently? We sat down and identified the root causes and factors affecting performance.* — District director of health

## CHALLENGES

During the discussions with the TPN members, it was noted that many of the challenges highlighted were not directly related to the twinning itself but rather to the priority issues that members had selected.

### Lack of Adapting Standards to Public and Private Hospital Structure Differences

The twinning approach should be a channel to inform the challenges that exist in hospitals. Some standards were not equally applied to public hospitals as they were applied to private hospitals [i.e., community need assessment]. A quality improvement officer of a private facility noted that private hospitals do not have a specific catchment area, whereas public hospitals have a specific district they cover, but the way the standard was measured during the accreditation survey remained the same.

### Lack of Integration of Twinning Into District Performance Contracts for Its Sustainability

Participants in the TPN recognize the approach as a valuable tool, not only for addressing technical and operational challenges but also as a strategic opportunity that should be embedded within the broader health system frameworks. Many believe that the sustainability of the twinning model relies on its formal integration into existing structures, such as district performance contracts, to ensure it becomes a key element of long-term health system strengthening.

*The twinning approach was a tool for us technical persons to advocate at [MOH], district authorities and province on our daily challenges in area of low performances which are out of our control and requiring multilayer interventions to get things done. We recommend the MOH add this approach to performance [contracts]of the DHMT and districts or MOH to ensure the sustainability of the twinning.* —District director of health

### Projected Population Did Not Reflect the Current Situation in Districts

Many DHMT members recognize that the target population (expected number of people to be enrolled in CBHI) is still a challenge, given that statistics are not updated annually.

*When you know that everyone in the community has paid for their membership but when the report comes out and you find out that you still have many households/individuals that are expected, yet you know that in reality, everyone is covered, then you feel discouraged and not sure what to do next.* —District director of health

### Implementation Challenges

- The COVID-19 pandemic impacted twinning implementation. Many of the activities were initially designed to be in person, but during the pandemic, some activities switched to virtual, which limited contact between districts and hospitals.
- During the pandemic, we found that participants were not as engaged in activities that switched from in person to virtual. Poor Internet network coverage in some areas, attendees’ ability to handle technology, and other work distractions were common challenges.
- Throughout implementation, TPN member staff from some districts and hospitals experienced staff turnover or change in positions—people switched jobs, left the institutions, and transferred to other departments or working places—which impacted twinning activities.
- TPN member staff have a lot of work; they often had no choice but to focus on other priorities, which impacted the way twinning activities were implemented, sometimes causing delays or cancellations.
- The project was not able to support all DHMTs to conduct the exchange visits due to budget constraints. However, when representatives from districts facing issues in their priority area visited districts excelling in that area, they testified that it added value far more than attending a virtual experience-sharing event.
- For some TPN member staff, changes in priority areas caused challenges in organizing and facilitating individual contact between twinning partners, especially when the other party and network facilitators were not informed about the intention to change the priority area.

## LESSONS LEARNED

Although twinning may be applied in many contexts, we learned that some considerations make twinning partnerships more appropriate in certain contexts than others. Partner institutions that work in similar settings and have similar roles and responsibilities and, therefore, share common goals made the implementation of a twinning partnership easier and more beneficial. Other lessons learned throughout the implementation of the twinning partnership include the following.

Partner institutions that work in similar settings and have similar roles and responsibilities and therefore share common goals made the implementation of a twinning partnership easier and more beneficial.

### Support of District and Hospital Senior Leadership

Senior leadership’s support and ownership aids in achieving twinning partnership objectives. When leadership was involved, staff were more motivated and felt empowered to achieve the twinning objective. District and hospital leaders also provided resources (human and/or financial) to accomplish the twinning goal when they were actively involved, committed, and aware of the learning potential, as well as what the twinning partnership was and how it could benefit their institution.

### Selection of the Right Twinning Partner

The success of twinning partnerships relied on institutional willingness to adapt and apply new processes and initiatives learned from their peers. Selecting the right twinning partner—one that has the capacity to transfer skills and experience as well as share knowledge—was critical in keeping partner institutions engaged in achieving the twinning goals.

### Exchange Visits Strengthen Partnerships

Many members of the TPN attributed value to the exchange visits. During the visits, both teams devoted time and attention to the process, learned through exchanging ideas, and evaluated the applicability of new techniques. Interviewees called exchange visits catalysts for change, inspiring teams to implement new initiatives and processes aimed at improving the quality of their health services.

### Limitations

We attempted to isolate the performance improvements by comparing intervention and nonintervention districts and hospitals over the same period but were unable to randomize the intervention districts and hospitals; rather, districts and hospitals self-selected focus areas. As a result, certain biases from self-selection cannot be excluded, including differences in intrinsic motivation, leadership skills, staffing patterns, and resources. Additionally, we report accreditation scores and CBHI enrollment from secondary data. As a result, we relied on the data from the Government of Rwanda to be accurate and represent actual changes in performance and enrollment. After a review, we believe the methodology is robust and the data are accurate.

In addition, there are also limitations imposed by using in-depth interviews of participants to understand the TPN’s impact, including recall bias, social desirability bias, and researcher bias. We attempted to control for recall bias by holding interviews concurrently with the final coaching visits. It was difficult to control social desirability and researcher bias because the interviewers were the same people who had developed and implemented the TPN, leading to some level of familiarity between the interviewers and interviewees. Resource constraints prevented separating these roles.

Interviews were conducted in Kinyarwanda, but the notes were taken in English. This method relied on immediate translation by the note-taker rather than a separate, thorough translation step. As a result, there is a possibility that some information may have been missed or inaccurately translated, which cannot be verified through a secondary translation process. This potential for translation inaccuracies may have affected the completeness and reliability of the data.

## CONCLUSIONS

The TPN is a platform through which districts and hospitals learn and share experiences, skills, and best practices with their peers and has contributed to significantly improving performance in hospital accreditation and CBHI enrollment. Qualitatively, participants appreciated the TPN as an opportunity to collaborate and network, strengthen and institutionalize systems, and identify factors and gaps in health systems that affect performance. This acknowledgment of both the quantitative and qualitative outcomes underscores the TPN’s role in fostering meaningful partnerships and enhancing health system performance through shared learning and support. We acknowledge a major challenge with institutionalizing the TPN within the structure of the Rwanda health system. Despite significant efforts to institutionalize the model, sustainability is not assured until the MOH both embeds the model into DHMTs performance reviews to foster long-term impact and funds districts and hospitals to participate in cross-district learning.

## Supplementary Material

23-00280-Gasana-Supplements.pdf

## Notes

**Peer Reviewed**

**First Published Online:** October 9, 2024.
