# Supplementary material for: Twinning Partnership Network: A Learning and Experience-Sharing Network Among Health Professionals in Rwanda to Improve Health Services
Source: Glob Health Sci Pract. 2024 Oct 29;12(5):e2300280. doi: 10.9745/GHSP-D-23-00280 (PMC11521555; doi:10.9745/GHSP-D-23-00280)
Supplement: 23-00280-Gasana-Supplements.pdf [file 23-00280-Gasana-Supplements.pdf]

Supplement to: Gasana C, Twinning partnerships network: a learning and experience-sharing network among health professionals in Rwanda to improve health services. *Glob Health Sci Pract*. 2024;12(5):e2300280. https://doi.org/10.9745/GHSP-D-23-00280

Supplement 1. Recommendations for the selection of twinning participants

Based on the Rwanda TPN, this paper recommends implementing a set of criteria for the selection of participant institutions and staff to enhance transparency and effectiveness in twinning partnerships. Suggested criteria include:

1. Institutional readiness: assess the institution's capacity and commitment to participate actively in the twinning process.
2. Staff expertise: select staff with relevant skills and capacity to transfer skills.
3. Clear roles and responsibilities: define and communicate the roles and responsibilities of each participant to avoid misunderstandings and ensure accountability.
4. Equitable representation: strive for a balanced representation of diverse perspectives and expertise to foster mutual benefit and learning.

Supplement to: Gasana C, Twinning partnerships network: a learning and experience-sharing network among health professionals in Rwanda to improve health services. *Glob Health Sci Pract*. 2024;12(5):e2300280. https://doi.org/10.9745/GHSP-D-23-00280

Supplement 2 Table S1. DHMT priority areas

Priority area	Rising district	Shining district
1. Improve accreditation scores	Bugesera	Rwamagana
	Gicumbi	Rwamagana
	Gakenke	Karongi
	Rulindo	Ngoma
	Kayonza	Gasabo
	Nyagatare	Ngoma
	Gatsibo	Ngororero
	Nyarugenge	Rwamagana
	Muhanga	Rwamagana
	Nyamasheke	Ngoma
	Rusizi	Ngororero
2. Increase CBHI coverage	Musanze	Gakenke
	Rwamagana	Gakenke
	Gasabo	Gicumbi
	Kicukiro	Nyaruguru
	Rutsiro	Gisagara
	Ngororero	Ruhango
	Rubavu	Gicumbi
	Nyabihu	Nyaruguru
3. Improve antenatal care	Ngoma	Kayonza
	Kirehe	Gakenke
	Kamonyi	Kayonza
	Nyanza	Gakenke
	Huye	Muhanga
	Nyamagabe	Gisagara
	Karongi	Kayonza
4. Increase family planning	Burera	Nyanza
5. Reduce home deliveries	Ruhango	Muhanga
6. Reduce stunting among children under 5	Nyaruguru	Nyamagabe
7. Reduce malaria incidence	Gisagara	Rusizi

Supplement to: Gasana C, Twinning partnerships network: a learning and experience-sharing network among health professionals in Rwanda to improve health services. *Glob Health Sci Pract*. 2024;12(5):e2300280. https://doi.org/10.9745/GHSP-D-23-00280

Supplement 2 Table S2. Hospital priority areas

**Priority area**	**Rising hospital**	**Shining hospital**
1. Improve hospital Accreditation	Butaro Hospital	Kibuye Referral Hospital
	La Croix du Sud Hospital	Kibuye Referral Hospital
	Ruhengeri Referral Hospital	Kibungo Referral Hospital
2. Improve postpartum family planning	Kacyiru Hospital	Kibagabaga Hospital
	Kibuye Hospital	Ruhango Hospital
3. Decrease stock-outs	Kibagabaga	Ruhengeri Referral Hospital
	Kibungo Referral Hospital	Ruhengeri Referral Hospital
4. Improve management of and care for birth asphyxia	Kabutare Hospital	Kacyiru Hospital
5. Improve laboratory quality control	Ndengera Polyclinic	Kibuye Referral Hospital
6. Reduce CBHI billing discrepancies	Ruhango Hospital	Kibuye Referral Hospital

Supplement to: Gasana C, Twinning partnerships network: a learning and experience-sharing network among health professionals in Rwanda to improve health services. *Glob Health Sci Pract*. 2024;12(5):e2300280. https://doi.org/10.9745/GHSP-D-23-00280

Supplement 3. Discussion guide

1. In thinking about your DHMT's or hospital's progress toward achieving the Twinning Partnership Plans:
   a. Did you achieve your twinning objectives?
   b. What performance improvements did your DHMT or hospital achieve over the life of the TPN?
   c. For CBHI enrollment and accreditation, please identify what changes were made and provide performance data.

2. What challenges prevented your DHMT or hospital from achieving the targets identified in the Twinning Partnership Plans?
   a. What could be done in future to overcome these challenges?

3. What lessons did your team learn about how to make twinning more effective?

4. What next steps would you recommend for implementing the TPN?

5. What did you appreciate most through the twinning partnership?

Supplement to: Gasana C, Twinning partnerships network: a learning and experience-sharing network among health professionals in Rwanda to improve health services. *Glob Health Sci Pract*. 2024;12(5):e2300280. https://doi.org/10.9745/GHSP-D-23-00280

Supplement 4 Table S1. Hospital accreditation performance scores (level 2) for TPN member hospitals that selected improving accreditation scores as their priority area

Hospital	Score by year (in %)		Difference (%)
	2021	2023	
Rwinkwavu	56	86	30
Byumba	64	80	16
Kabgayi	46	75	29
Kibogora	59	48	-11
Nemba	59	55	-4
Ngarama	55	80	25
Mibilizi	42	50	8
Nyagatare	55	68	13
Kinihira	48	60	12
Gahini	61	81	20
Ruli	58	52	-6
Nyamata	54	78	24
Rutongo	58	63	5
Gihundwe	67	42	-25
Muhima	65	69	4
Ruhengeri	82	83	1
Bushenge	77	73	-4
Kiziguro	72	81	9
Butaro	77	80	3
**Average**	**60.8**	**68.6**	**7.8**

Supplement to: Gasana C, Twinning partnerships network: a learning and experience-sharing network among health professionals in Rwanda to improve health services. *Glob Health Sci Pract*. 2024;12(5):e2300280. https://doi.org/10.9745/GHSP-D-23-00280

Supplement 4 Table S2. Hospital Accreditation performance scores (level 2) for non-TPN member hospitals

Hospital	Score by year (in %)		Difference (%)
	2021	2023	
Ndera	39	74	35
Kirehe	62	93	31
Shyira	73	80	7
Muhororo	86	88	2
Kigeme	83	94	11
Kabaya	79	77	-2
Masaka	77	88	11
Kaduha	69	44	-25
Kibilizi	63	79	16
Murunda	60	77	17
Gitwe	44	75	31
Mugonero	86	92	6
Remera-Rukoma	67	55	-12
Kirinda	69	74	5
Munini	77	55	-22
Gakoma	82	56	-26
Rwamagana	92	71	-21
Gisenyi	73	74	1
Nyanza	79	56	-23
Average	71.6	73.8	2.2

Supplement 4 Table S3. Hospital accreditation performance scores (Level 2) for TPN member hospitals that selected priority areas other than improving accreditation scores

Hospital	Score by year (in %)		Difference (%)
	2021	2023	
Kibagabaga	23	83	60
Kabutare	36	62	26
Kibuye	71	82	11
Kacyiru	85	88	3
Ruhango	67	74	7
Kibungo	84	85	1
**Average**	**61**	**79**	**18**

Supplement to: Gasana C, Twinning partnerships network: a learning and experience-sharing network among health professionals in Rwanda to improve health services. *Glob Health Sci Pract*. 2024;12(5):e2300280. https://doi.org/10.9745/GHSP-D-23-00280

Supplement 4 Table S4. CBHI coverage in CBHI priority districts

District	2021, %	2023, %	% Increase
Kicukiro	75.2	95.1	19.9
Ngororero	83.4	91.2	7.8
Rutsiro	78.2	86	7.8
Gasabo	80.4	88	7.6
Rubavu	84.7	87.6	2.9
Rwamagana	84.1	85.5	3.2
Nyabihu	82.9	87.3	2.6
Musanze	81.9	83.2	1.3
**Average**	**81.4**	**88**	**6.6**

Supplement 4 Table S5. CBHI coverage in non-CBHI priority districts

District	2021, %	2023, %	% Change
Nyarugene	80.2	93	12.8
Rusizi	84.5	91.2	6.7
Karongi	84.9	91.2	6.3
Nyamasheke	87.8	94.1	6.3
Gicumbi	90	94	4
Nyaruguru	92	95.9	3.9
Rulindo	85.3	89.1	3.8
Kayonza	84.7	88.5	3.8
Gisagara	95	98.4	3.4
Kirehe	88.3	91.7	3.4
Gakenke	92.9	95.1	2.2
Burera	87	89.2	2.2
Bugesera	83.9	85.8	1.9
Gatsibo	80.5	82	1.5
Nyamagabe	91.5	93	1.5
Ruhango	91.3	92.7	1.4
Kamonyi	89	90.2	1.2
Nyanza	85.2	86.3	1.1
Huye	82.8	83.7	0.9
Ngoma	87	87.5	0.5
Nyagatare	79.5	79.5	0
Muhanga	88.7	86.5	-2.2
**Average**	**86.9**	**89.9**	**3**
